# Defense against territorial intrusion is associated with DNA methylation changes in the honey bee brain

**DOI:** 10.1186/s12864-018-4594-0

**Published:** 2018-03-26

**Authors:** Brian R. Herb, Molly S. Shook, Christopher J. Fields, Gene E. Robinson

**Affiliations:** 1 Carl R. Woese Institute for Genomic Biology, University of Illinois at Urbana-Champaign, Urbana, IL USA; 2 Department of Entomology, University of Illinois at Urbana-Champaign, Urbana, IL USA; 3 Neuroscience Program, University of Illinois at Urbana-Champaign, Urbana, IL USA

**Keywords:** Aggression, DNA methylation, Brain, Epigenetics, Transcription factor binding sites, Evolution

## Abstract

**Background:**

Aggression is influenced by individual variation in temperament as well as behavioral plasticity in response to adversity. DNA methylation is stably maintained over time, but also reversible in response to specific environmental conditions, and may thus be a neuromolecular regulator of both of these processes. A previous study reported DNA methylation differences between aggressive Africanized and gentle European honey bees. We investigated whether threat-induced aggression altered DNA methylation profiles in the honey bee brain in response to a behavioral stimulus (aggression-provoking intruder bee or inert control). We sampled five minutes and two hours after stimulus exposure to examine the effect of time on epigenetic profiles of aggression.

**Results:**

There were DNA methylation differences between aggressive and control bees for individual cytosine-guanine dinucleotides (CpGs) across the genome. Eighteen individual CpG sites showed significant difference between aggressive and control bees 120 min post stimulus. For clusters of CpGs, we report four genomic regions differentially methylated between aggressive and control bees at the 5-min time point, and 50 regions differentially methylated at the120-minute time point following intruder exposure. Differential methylation occurred at genes involved in neural plasticity, chromatin remodeling and hormone signaling. Additionally, there was a significant overlap of differential methylation with previously published epigenetic differences that distinguish aggressive Africanized and gentle European honey bees, suggesting an evolutionarily conserved use of brain DNA methylation in the regulation of aggression. Lastly, we identified individually statistically suggestive CpGs that as a group were significantly associated with differentially expressed genes underlying aggressive behavior and also co-localize with binding sites of transcription factors involved in neuroplasticity or neurodevelopment.

**Conclusions:**

There were DNA methylation differences in the brain associated with response to an intruder. These differences increased in number a few hours after the initial exposure and overlap with previously reported aggression-associated genes and neurobiologically relevant transcription factor binding sites. Many DNA methylation differences that occurred in association with the expression of aggression in real time also exist between Africanized bees and European bees, suggesting an evolutionarily conserved role for epigenetic regulation in aggressive behavior.

**Electronic supplementary material:**

The online version of this article (10.1186/s12864-018-4594-0) contains supplementary material, which is available to authorized users.

## Background

Experiences produce molecular responses in the brain that exert long-term influences on behavior, affecting the way individuals or groups respond to future circumstances [1]. For example, acute stress, including exposure to social stressors, can lead to a transient state of increased aggression [2]. This reaction to stress exists in many animal model systems, including honey bees. When faced with a territorial intruder, bees protect their colony with defensive behavior such as biting and stinging. Once disturbed, they develop a state of aggressive vigilance against other potential threats and exhibit heightened responses to future intrusions [3, 4].

Multiple observations motivate investigation into the epigenetics of this response. First, there are extensive differences in gene expression in the honey bee brain that are related to aggression [4–10], including between the highly aggressive Africanized honey bee (AHB) subspecies *Apis mellifera scutellata* and the less aggressive European honey bee subspecies (EHB) [5]. In addition, whole-genome profiling of 5-methylcytosine (5mC) has revealed differences in DNA methylation between AHB and EHB [9]. Although these results suggest the possibility that aggression is epigenetically regulated in honey bees, AHB and EHB differ in other traits besides aggression. The question of whether brain epigenetic profiles are associated specifically with aggressive behavior in bees therefore remains open.

There is a well-established relationship between behavioral plasticity and dynamic DNA methylation in several model systems. In mammals, modulation of epigenetic marks in the brain has been linked with exposure to social stressors, including separation of offspring from their mothers [11, 12] and chronic social defeat [13, 14]. Recent evidence indicates that DNA methylation underlies the maintenance of long-term memory [15–18], allowing experiences to stably alter behavior. The de novo addition and active removal of DNA methylation facilitate transitions between epigenetic states in the brain [19–22], suggesting a compelling molecular mechanism underlying behavioral plasticity.

Knowledge of genetic and social effectors of aggression in honey bees, coupled with a fully functional epigenetic toolkit [23], make honey bees an excellent organism to study the molecular basis of aggression. DNA methylation changes in the honey bee brain are associated with behavioral plasticity, most notably in switching between nursing and foraging roles in the hive division of labor [24]. Additionally, differences in brain DNA methylation have been reported between queen and worker bees during development and in adulthood when they display distinct behaviors [25, 26]. Early evidence for DNA methylation’s impact on queen / worker development was illustrated by DNMT knockdown during the larval stage that produced queens despite a feeding regime that typically produces workers [27].

Division of labor is a hallmark of eusocial species, and mounting evidence shows that DNA methylation plays a role in this process, possibly by affecting vitellogenin levels and altering lifespan [28]. In the brain, pharmacological perturbation of DNA methylation affects learning ability, and long-term memory formation appears to require upregulation of DNA methylation modifying enzymes DNMT and Tet [29]. This evidence, in addition to DNA methylation’s predicted role in regulating gene expression and alternative splicing events, makes DNA methylation an attractive candidate for regulating aggressive behavior in honeybees [30].

We used a well-established aggression assay to investigate the role of DNA methylation in mediating aggressive responses to an intruder [4]. Although other stimuli such as alarm pheromone also elicit defensive behavior, we used the intruder assay because it provokes strong aggressive responses that are easily quantified, including biting and stinging. To profile DNA methylation signatures associated with aggression in the brain, we performed whole genome bisulfite sequencing (WGBS) of DNA from the brains of honey bees that responded aggressively to territorial intrusion, and compared the DNA methylation profiles with those of control bees who experienced an inert stimulus. DNA methylation was profiled at early and late time points (5 and 120 min, 6 individuals per phenotype) following the intrusion to investigate epigenetic differences between aggressive and control bees.

Here we show DNA methylation profile signatures of aggressive behavior in honey bees co-localize with gene regulatory elements in *cis* and are reflected in differences between aggressive Africanized bees and gentle European bees. DNA methylation differences increase over time from an early state immediately after interaction with an intruder to two hours post exposure, which is when memory consolidation typically occurs in other experimental systems [31]. These epigenetic profiles thus correlate with other findings that relate to both evolutionary and physiological time scales and provide insight into the molecular mechanisms of aggression.

## Results

### Effects of stimulus on methylation levels of individual cytosines over time

Out of 3,897,088 CpG sites with an average coverage of at least 5X, we identified 119,557 methylated CpG sites where at least 3 out of 24 samples had DNA methylation levels greater than 10%. As previously reported in honey bees, most of the DNA methylation resided within exons [25]. We found that 97,245 of the methylated CpG sites resided within gene bodies, with 69,831 overlapping exons. Additionally, 25,190 CpG sites were within promoters 2 kb upstream of transcriptional start sites, some of which also overlapped gene bodies.

Individual t-tests were performed on arcsine transformed methylation percentages and subject to multiple testing correction (FDR < 0.05) to compare aggressive and control bees within each time point and across time. While no individual CpGs were significantly different between aggressive and control bees 5 min after stimulus, 18 CpGs distinguished aggressive and control bees 120 min post stimulus (FDR ≤ 0.05). Most (16/18) of these CpGs were clustered on genomic scaffold 2.11. This cluster included two TRP channel genes, GB50805 and GB50806, that could play a role in sensing environmental stimuli [32]. Additionally, there were two CpGs differentially methylated between 5-min and 120-min control samples, and no differences between 5-min and 120-min aggressive samples. These results provide evidence for methylation changes in the brain in response to intruder exposure with clear differences 120 min post stimulus.

### Regional analysis of DNA methylation reveals differences resulting from intruder exposure over time

Since the majority of differentially methylated CpGs co-localized to a region in scaffold 2.11, we next looked for additional regional methylation differences among clusters of CpGs and assessed significance at the regional level. This approach has been used to identify differences in cancer types [33], and it allowed us to directly compare aggressive individuals to control bees within each time point. We identified four differentially methylated regions (DMRs) between aggressive and control bees at the 5-min time point (Additional file 1: Table S1.). These DMRs could either indicate a very rapid epigenetic response to the intruder, or they might have existed prior to intruder exposure; akin to individual differences in social responsiveness previously reported in honey bees [34]. Some of these DMRs were located near or within genes that contain ion channel or receptor domains, with known neuronal functions in insects. One DMR within scaffold 8.9 is located over the 5′ end of NMDR3, an NMDA (n-methyl-D-aspartate) receptor, previously associated with increased aggression [35]. Other DMRs overlap with a homolog of CG42340, a regulator of synaptic plasticity in *Drosophila* [36].

**Fig. 1:**
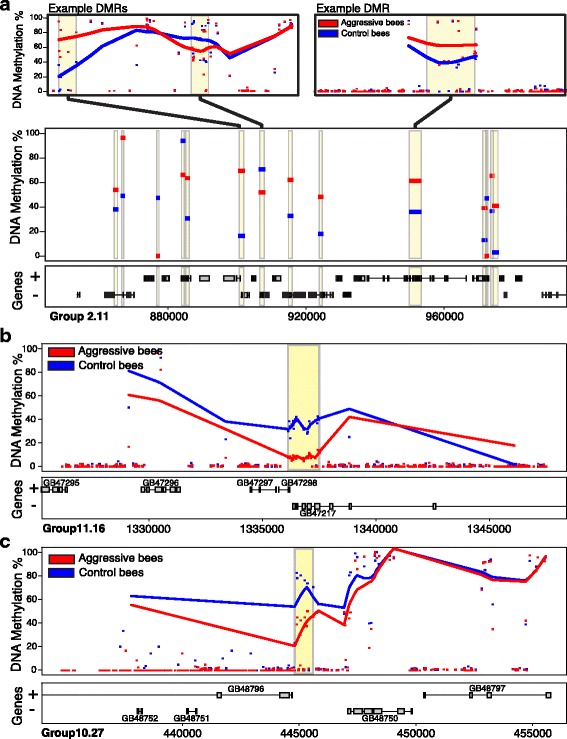
Regional DNA Methylation Differences Distinguish Aggressive And Control Bees. Aggressive bees (red) and control bees (blue) show distinct DNA methylation profiles. **a**) scaffold 2.11 contains 21 of the 50 min-120 DMRs. The region depicted spans the ~ 90 kb hotspot of DNA methylation change and contains many signaling genes. Top panel shows two examples of DMRs within scaffold 2.11 with smoothed lines representing average methylation levels of individual methylated CpG’s (ignoring CpGs with < 10% methylation levels). Middle panel displays “hotspot” region of scaffold 2.11 containing multiple DMRs. Horizontal bars within yellow DMR areas show average methylation levels for each DMR. **b-c**) Top panel presents DNA methylation level for a short segment of scaffold 11.16, with dots for individual CpGs and smoothed lines for average methylation levels for each phenotype. The DMR in (**b**) is located over the DH31-R (GB47217) calcitonin receptor, and the DMR in **(c)** is over the DH44 (GB48796) diuretic hormone gene, both important for stress response

At 120 min following intruder exposure, there were 50 DMRs between the aggressive and control bees (Additional file 2: Table S2.). This approximate ten-fold increase in the number of DMRs from 5 to 120 min, with no overlap between DMR sets, demonstrates sweeping temporal changes in the brain epigenetic response to an intruder. It is also consistent with the finding from Shpigler et al. (2017a), which performed a similar aggression study with honey bees and found the largest number of differentially expressed genes (DEGs) at 120 min after intruder exposure [4]. This DEG analysis also revealed no expression bias of DNA methyltransferase enzymes in either phenotype, which is reflected in the fact that we observe both hyper- and hypo- methylation within the 120 min DMRs. 120-min DMRs include most (11/16) CpGs from scaffold 2.11 that were identified independently, and confirm that this is a hotspot of epigenetic change, with 21 out of 50 DMRs residing on this scaffold (Fig. 1a). Increasing numbers of DMRs from 5 to 120 min also strengthens our finding of change over time within individual CpGs and strongly suggests that the DNA methylation changes are a direct response to intruder exposure.

Genes associated with 120-min DMRs include those involved with RNA processing, chromatin remodeling and hormone signaling, processes that have been previously implicated in brain transcriptomic analyses of the bee aggression [4, 7]. DMR-associated chromatin remodeling genes included the tudor domain containing spoon and SET domain binding factor Sbf. There were also highly significant DMRs associated with DH44 (diuretic hormone 44) and DH31-R (calcitonin receptor), which are evolutionarily related to proteins in the mammalian CRF (corticotropin-releasing factor) and CGRP (calcitonin gene-related peptide) stress hormone signaling pathways (Fig. 1b-c). In vertebrates, CRF and its receptors have been identified as regulators of aggression and responses to social stress [13]. Overall, genes associated with 120-min DMRs may help facilitate large-scale gene expression response to intruder and alter signaling pathways that heighten the response to a new intruder in the future, because the presence of one intruder often presages the presence of more [37].

### Correlation analysis utilizing individual cytosines suggests a time-dependent impact on gene expression and an enrichment of transcription factor binding sites

Since other forms of behavioral plasticity that involve neuronal remodeling, such as memory formation, require coordinated gene expression changes, we investigated the potential impact of DNA methylation on gene regulation across the genome. On the cellular level, DNA methylation is known to play a critical role in the late-phase of long-term potentiation (L-LTP, ~ 2 h post induction), when gene expression changes stabilize the strengthening of synapses [27]. To explore whether DNA methylation might work on a similar time scale in the context of aggression, we related the present results to previously published transcriptomic profiles in Shpigler et al. (2017a), wherein gene expression profiles were made at 30, 60, and 120 min after intruder exposure in the mushroom bodies (MB), rather than the whole brain [4] (The MB in honey bees are very prominent and constitute ca. 40% of total brain volume [38]). In this exploratory analysis, we checked the overlap of the DEGs from Shpigler et al. (2017a) with suggestive individual CpGs in the present study that passed a relaxed statistical threshold (t-test *p*-value < 0.05, but not subject to FDR correction). This relaxed threshold identified 3689 suggestive CpGs that were differentially methylated between aggressive and control bees 5 min post exposure, and 4323 CpGs 120 min post exposure. These suggestive CpGs in aggregate distinguish aggressive and control bees on a genome-wide level, but cannot be thought of in this way when considering any individual CpG. They were used to compare our findings to other genome-wide datasets. All conclusions made using suggestive CpGs were evaluated on the genome-wide level, not on the individual CpG level.

**Fig. 2:**
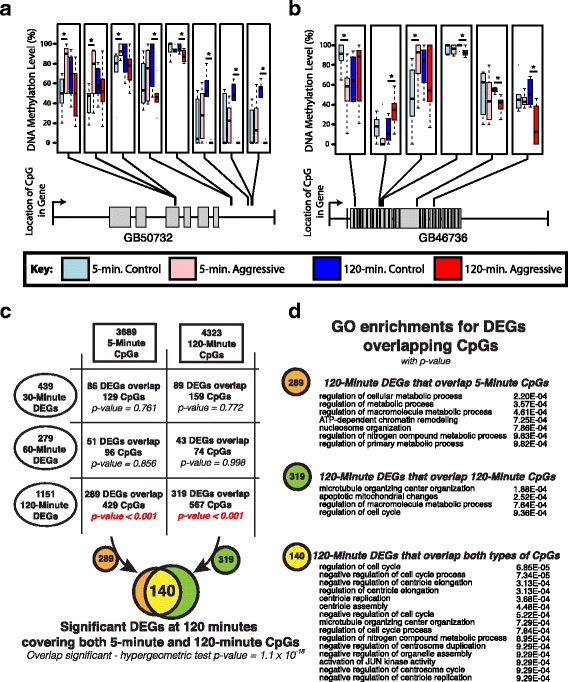
Gene expression changes in response to intruder are marked by both early and late epigenetic differences. Gene expression differences between aggressive and control bees that arise 120 min after interaction with an intruder are often marked with differences in DNA methylation. There were 140 genes that had differential methylation both before and after gene expression changes occurred. For both (**a**) and (**b**), the top panel depicts boxplots of DNA methylation levels at both time points for aggressive and control bees. Asterisks indicate significant differences between aggressive and control bees within time points. Note that different sets of CpGs have significant differences at different time points, thus marking gene before and after gene expression change at 120 min with distinct CpGs. The bottom panel shows the differentially expressed gene and the location of the CpGs within the gene body. (**c**) Summary of overlap between 120-min DEGs and suggestive CpG’s. Both 5-min and 120-min suggestive CpGs have significant overlaps with120-minute DEGs, with a high degree of co-localization within a subset of genes. (**d**) GO enrichments for genes both differentially expressed and differentially methylated in response to intruder exposure

This analysis revealed that only 120-min DEGs have a significant overlap with suggestive CpGs (both *P*-value < 0.001 based on 1000 permutations, Additional file 3: Figure S1 a-f). Surprisingly, both 120-min and 5-min CpGs have significant overlaps with 120-min DEGs, often co-localizing in the same gene (Fig. 2, full GO annotation results in Additional file 4: Table S3). A total of 289 120-min DEGs overlapped 5-min CpGs and 319 120-min DEGs overlapped 120-min CpGs. We also observed a significant co-localization of 120-min and 5-min suggestive CpGs in 140 of the 120-min DEGs (overlap *p* = 1.1 × 10^− 18^ by hypergeometric test). Although 120-min DEGs overlapped both 5-min and 120-min CpGs, these CpG groups were largely distinct, with only 11 common CpGs within 120-min DEGs across time points. Together, these findings reveal a significant number of methylation differences marking 120-min DEGs, both immediately after and 2 h post exposure to an intruder. Existence of differential methylation before gene expression change might poise a set of genes ready to respond, much like bivalent epigenetic marks help poise genes in mammalian stem cells to respond to developmental cues [39]. Here, we found ~ 12% (140/1151) of 120-min DEGs marked by suggestive CpGs at 5 min were replaced by other suggestive CpGs at 120 min, potentially switching from a poised state to locking in the gene expression change post exposure.

### DMRs and suggestive CpGs overlap DNA methylation between Africanized and European honey bees, suggesting an evolutionary conserved role for epigenetics in regulating aggression

We explored whether the methylation differences we found to be associated with a real-time aggressive response were also associated with differences in aggression that occur on an evolutionary time scale. Evolutionary differences in colony-level aggression exist between European and African-derived subspecies of honey bees. Africanized honey bees (AHB) have a lower threshold needed to trigger an aggressive response and a larger proportion of aggressive individuals within a colony than do European honey bees (EHB), and Alaux et al. [5] found genes that differ in brain expression between AHB and EHB. Moreover, there was a statistically significant overlap between the genes that differed in expression between AHB and EHB and those that differed in EHB in response to exposure to alarm pheromone, which is associated with territorial intrusion and aggressive responses. We compared our within-colony (EHB) methylation differences to previously published methylation differences distinguishing AHB and EHB colonies [9]. We found a highly significant overlap between 120-min DMRs (and suggestive CpGs) and AHB / EHB differences (both *P*-value < 0.001 based on 1000 permutations, Additional file 3: Figure S1 g-h). This overlap suggests a conserved role for DNA methylation in modulating aggressive behavior across genetic backgrounds.

### DNA methylation co-localizes with TFBS that regulate neuroplasticity and neurodevelopment

To further understand the molecular role of DNA methylation in gene regulation, we assessed the co-occupation of transcription factor binding sites (TFBS) and suggestive CpGs within promoters across the genome. DNA methylation can directly block TFBS in promoters, as seen in mammalian systems [40]. To identify transcription factors that might interact with DNA methylation to mediate the transcriptional response to an intruder, we calculated hypergeometric tests for the overlap of putative TFBSs and 120-min suggestive CpGs within promoters (2 kb) as defined by the OGS 3.2 gene set (Additional file 5: Table S4). Fourteen TFBSs had a highly significant overlap with suggestive CpGs (FDR of < 1.5 × 10^− 10^), and eleven of these TFBSs have some previously reported role in either neuroplasticity or neurodevelopment. Among these were Hr51 and foxo, both of which were identified as part of a brain transcriptional regulatory network constructed for honey bee aggressive behavior [4]. Hr51 and foxo, along with other enriched transcription factors lola and jigr1 (via interaction with jing) all contribute to neuron remodeling in *Drosophila melanogaster* by assisting either axon guidance or dendrite branching [36, 41–43]. Other enriched transcription factors help shape brain morphology during development and may play a role in adult behavior, including fkh, vvl/dfr, CG10267/Zif and Optix [44–47]. Because many genes overlap, we also present the enrichment for the non-overlapping portion of the promoter in Additional file 5: Table S4. Outside of the promoter, we observed strong enrichment of DNA methylation and TFBS within genes, potentially serving a protective role against spurious TF binding in gene bodies, as observed in termites [48]. Across the genome, we also observed that TFBS of Cf2, CG8281, jigr, lola, fkh, vvl, and CG10267/Zif were enriched over 120-min suggestive CpG sites by AME analysis [49]. For these TFBS enriched over suggestive CpGs, DNA methylation may be acting directly and altering the binding affinity for the TF. While largely unexplored in insects, DNA methylation might play a similar role in affecting transcription factor binding as in mammals [50].

## Discussion

We present, to our knowledge, the first DNA methylation profiles of aggression in response to territorial intrusion. Our choice of honey bees as a model organism allowed us to directly study known aggressive individuals within a social context and their molecular response to an intruder. We found far more methylation differences between aggressive and control bees at 120 min than at 5 min after intruder exposure, which implies that DNA methylation could be assisting in altering neurogenomic states to maintain an aroused and vigilant state. Intruder-induced increases in arousal over the time scale used in this study are well known in the animal world and have been documented in honey bees [4, 5]. The observed brain epigenetic differences had a significant overlap with both gene expression differences in response to an intruder and with DNA methylation differences across genetic strains of honey bees that differ drastically in response to intruders.

Honey bees exhibit an increased sensitivity to future territorial intrusions after an initial interaction with an intruder in the colony [3, 4]. A change in response threshold to subsequent intruder interactions likely requires neuronal remodeling and has been shown to involve gene expression changes in genes related to steroid hormone receptor activity and chromosome organization [4]. Epigenetic mechanisms provide a flexible means of gene regulation that can result in temporary gene expression changes and contribute to neural plasticity. This hypothesized link between epigenetic regulation and neural plasticity is further strengthened by the highly significant overlap we found between DNA methylation differences and binding sites for neurobiologically relevant transcription factors. This evidence suggests a mechanism whereby DNA methylation can affect brain gene regulation over a very short time period by blocking or enhancing the action of transcription factors.

We also provide evidence for DNA methylation marking genes prior to expression changes, thus potentially poising genes in a ready state that can more immediately respond to an encounter with an intruder. Additionally, we previously reported differences in histone H3 lysine 27 acetylation (H3K27ac), a marker of open chromatin, in mushroom bodies at the same time point after intruder exposure [4], further supporting the conclusion that aggression leads to epigenetic reconfiguration in the brain. Interestingly, neither our DMRs nor suggestive CpGs overlapped regions of differential H3K27ac from Shpigler et al. (2017a), possibly suggesting a different role for these two epigenetic modifications. Lastly, intruder exposure also rapidly alters H3K27ac levels in the diencephalon of freshwater stickleback fish, suggesting an evolutionarily conserved role for epigenetic response to and intruder [51].

There is growing interest in exploring the link between epigenetics, early life stressors and altered behavior. In humans, multiple genes including IL-6 interleukin 6 and the serotonin transporter SLC6A4 (solute carrier family 6 member 4) have altered DNA methylation levels in their promoter regions (reviewed in Waltes et al. [52]) thought to be caused by early life trauma. Model organisms such as rats and chickens have provided direct evidence of artificially elevated levels of cortisol or diminished maternal care early in life leading to altered epigenetic levels of key genes involved in aggressive behavior. Our study adds to this growing literature especially with the finding of dynamic changes in brain methylation associated with aggression-related genes after exposure to an intruder. These changes suggest epigenetic involvement in altering future aggression-related behavior.

## Conclusions

Behavioral plasticity allows organisms to adapt to new adverse circumstances, including hostile territorial intrusions. We showed that differences exist between the brain DNA methylation profiles of aggressive and control honey bees, indicating a possible epigenetic basis for plasticity in aggression. Most of the differences were observed two hours after exposure to a foreign intruder but not at an earlier time point, suggesting that they arose as a direct result of experience. Additionally, we detected a few methylation differences between aggressive and control bees five minutes after intruder exposure, possibly reflecting pre-existing individual differences in the epigenetic control of aggressive tendencies. This study provides the first evidence that changes in brain DNA methylation occur in response to territorial intrusion, supporting the conclusion that there is an epigenetic basis to behavioral plasticity in aggression.

## Methods

### Laboratory intruder assay and bee collection

Honey bee workers used in this study were derived from a queen instrumentally inseminated with semen from a single drone and reared up to adulthood in a colony maintained under typical conditions at the University of Illinois at Urbana-Champaign Bee Research Facility, Urbana, Illinois. Bees in this area are a mixture of European subspecies of *Apis mellifera*, primarily *ligustica*. Honeycomb frames containing older pupae were collected from a single colony and maintained in an incubator room at 34 °C, 50 ± 5% humidity. Emerging adults were collected every 24 h and marked on the thorax with Testor’s paint. The bees were placed in groups of ten in vertically oriented petri dishes lined with a layer of beeswax foundation [4]. They were fed 30% sucrose solution and pollen balls made of fresh frozen pollen mixed with 30% sucrose solution. Food was checked daily and replenished as necessary. The groups were kept in the incubator room for 8 days to establish group identity prior to the intruder assay.

Aggression was assessed using a laboratory intruder assay modified from [4], which includes a supplemental video of the assay. A foreign worker bee from another colony was introduced into each petri dish as an intruder, and biting and stinging responses to the intruder were observed and recorded for the next 5 min. The colored paint dots on the bees allowed for assignment of each aggressive event to an individual bee. The two most aggressive bees, defined as those displaying the highest number of biting and stinging events, were collected from each group and flash frozen in liquid nitrogen. Collections were done either 5 min or 120 min following initial intruder exposure. Control groups were exposed to a 0.2 ml plastic tube similar in size to a worker bee, which was removed after 5 min. Two bees were collected from each control group at random either 5 min or 120 min following initial exposure to the tube. After flash freezing, all collected bees were transferred individually to microfuge tubes and stored at − 80 °C.

### Sample preparation and whole-genome bisulfite sequencing

The heads were removed from frozen bees and placed into a dry ice/100% ethanol bath. The frons were removed with a scalpel and the heads were completely submerged in RNALater ICE overnight at − 20 °C, allowing the reagent to permeate the brain tissue for 15 to 16 h. Whole brains were dissected from head capsules at room temperature using a scalpel and fine forceps, with care taken to remove the adjacent hypopharyngeal gland. Individual brains were placed in microfuge tubes and stored at − 80 °C until DNA extraction.

Genomic DNA was extracted from whole brains using the Gentra Puregene Tissue Kit (Qiagen) and stored at − 20 °C. Unmethylated lambda phage DNA (Promega) was spiked in as a control for assessment of bisulfite conversion efficiency. The samples were given to the Roy J. Carver Biotechnology Center at the University of Illinois for quality control, library construction, and sequencing. The integrity of the samples was checked using the AATI Fragment Analyzer, which confirmed that the DNA was of high molecular weight. Shotgun DNA libraries were made using the KAPA Library Preparation Kit, then bisulfite treated using the Zymo EZ DNA Methylation-Lightning Kit. The libraries were then amplified for 10 PCR cycles using HiFi Uracil+ DNA Polymerase (KAPA Biosystems), pooled in equimolar quantities, and sequenced on the HiSeq 2500 (Illumina). Paired-end 100-nucleotide sequence reads were generated from six control and six experimental samples for each collection time point (5 min and 120 min). FASTQ files were generated and demultiplexed with the bcl2fastq v2.17.1.14 Conversion Software (Illumina).

### Alignment

Paired-end reads were trimmed with the trimmomatic program (v 0.33) using the following parameters: (ILLUMINACLIP:TruSeq3-PE-2.fa:2:15:10 CROP:98 HEADCROP:10 LEADING:20 TRAILING:20 SLIDINGWINDOW:4:15 MINLEN:30). Trimmed reads were aligned to the Amel_4.5 honey bee genome assembly with bowtie2 within the bismark program (v 0.16.1) using bowtie2 default settings except for max insert size set to 1000. The bismark program was also used to mark duplicates and extract DNA methylation levels for each CpG. The R package bsseq was used to merge DNA methylation information across all samples and calculate coverage. Bisulfite conversion efficiency was estimated both empirically by assessing number of unconverted cytosines that aligned to the lambda genome from the lambda spike-in control and assessment of typically unmethylated cyotsines across the genome using the MethPipe software [53] (Details: Additional file 6: Table S5).

### Differential methylation analysis at individual cytosines

Cytosines that had an average coverage across all samples of 5 or more reads and displayed at least 10% methylation levels in three or more individual bees were defined as methylated (119,557 met this criteria). We chose a minimum of three individuals to avoid spurious methylation in one or two samples while at the same time allow for CpG sites unique to sub-phenotypes that might only be represented by a small subset of bees. To find differences in DNA methylation between aggressive and control bees over time, we first arcsine transformed percentage measurements derived from ratios of reads containing methylated and unmethylated cytosines in the CpG dinucleotide context. Student’s t-test was performed on individual CpG sites and these raw *p*-values were used to identify suggestive CpGs (p-value ≤0.05) used in subsequent methods. Multiple test correction was performed using the qvalue package in R [54, 55] and a FDR was calculated for each methylated CpG.

### Identifying differentially methylated regions based on individual cytosines

To determine whether aggression is associated with changes in methylation at the regional level, we implemented a pipeline for the identification of differentially methylated regions (DMRs). Raw p-values for individual CpG sites within each region designated by the bumphunter package were combined using the *comb-p* software [56] and regional *p*-values were corrected for multiple testing. The comb-p package first calculates the correlation between proximal *P*-values of varying distances. The Stouffer–Liptak–Kechris correction (slk) is then applied which corrects each P-value respective of weighting determined by correlation calculation. This analysis identified regions of coordinated change where multiple CpG sites change in the same direction and pairwise comparisons were made between each combination of time point and stimulus (*n* = 6 for each group).

### Overlap of differential methylation and genomic features / published data

To assess the genome-wide impact of brain DNA methylation on brain gene expression, we used gene expression data from the Shpigler et al. (2017a) [4] study that utilized the same experimental set-up to identify aggressive bees. This study identified brain gene expression differences between aggressive and control bees at 30, 60, and 120 min after the introduction of an intruder bee to a petri dish containing test bees. To identify trends, we included all CpGs with a p-value ≤0.05 in our analysis (suggestive CpGs), which includes 3689 differences at minute-5 and 4323 at minute-120. The suggestive CpGs identified at each time point were largely distinct, with only 135 common CpGs across time points. We quantified the number of DEGs that overlap suggestive CpGs for each time point. To test the significance of this overlap, 1000 permutations of random sets of genes matching the number of significant DEGs were created for each time point and checked for overlap with suggestive CpGs. Empirical *p*-values for overlap were reported based on permutation results. Significance of overlap between 5-min and 120-min suggestive CpGs within 120-min DEGs was calculated by hypergeometric test.

A similar analysis was performed on DNA methylation data from Cingolani et al. [9], however, WGBS data was originally aligned to the Amel2 version of the genome, which necessitated alignment to the Amel4.5 version of the genome to compare results. Permutation analysis was used to calculate the degree of overlap between DMRs and suggestive CpGs found in our study to CpGs that differed between Africanized and European honey bees by 30%.

### Co-localization of DNA methylation and TFBS in promoters

Locations of putative transcription factor binding sites (TFBS) were found by mapping TFBS motifs identified in *Drosophila melanogaster* to the Amel 4.5 version of the honey bee genome using the program CisGenome [40]. Suggestive CpGs identified at minute-120 and TFBS were quantified within each promoter (2 kb upstream of TSS) for genes of OGS 3.2. Hypergeometric tests were performed to calculate *p*-values for overlap between DNA methylation and TFBS within promoters, and FDRs were calculated from p-values using the qvalue package in R [54, 55]. Additional analysis of genome-wide enrichment of TFBS around suggestive CpGs irrespective of gene location was performed using the AME program [49], which is part of the MEME suite. For each suggestive CpG identified at 120-min post intruder exposure, 20 base pairs of sequence up and down stream of cytosine was recorded (total 41 bp for each suggestive CpG). Background sets were based on the sequence surrounding 4000 randomly selected methylated CpGs (also 41 bp per cytosine). Target sequences were analyzed for enrichment of TFBS (Combined Drosophila Databases) against background sets.

## Additional files

Additional file 1: **Table S1.** DMRs between control and aggressive bees at 5 min post interaction. (XLSX 36 kb)

Additional file 2: **Table S2.** DMRs between control and aggressive bees at 120 min post interaction. (XLSX 61 kb)

Additional file 3: **Figure S1.** Results of permutation testing for DEG/CpG overlap and comparison with Cingolani et al. 2013 [9]. (a-f) Minute 5 and Minute 120 suggestive CpGs were overlapped with gene expression differences of Aggressive and Control bees from Shpigler et al. (2017a) [4]. To test the significance of the overlap, we chose at random the same number of significantly different DEGs, checked the overlap and repeated this process 1000 times. For each plot, the blue histogram represents the distribution of random trials and the red vertical line represents the true number of overlapping DEGs with suggestive CpGs. Note that both minute 5 and minute 120 suggestive CpGs had a highly significant overlap with only minute 120 DEGs. (g-h) A similar approach was taken to test the significance of the overlap between AHB / EHB differences and minute 120 DMRs and suggestive CpGs. In both cases the true overlap was greater than the 1000 permutations. (PDF 478 kb)

Additional file 4: **Table S3.** Details of GO enrichments for Differentially expressed genes from Shpigler et al. 2017a [34] that overlap suggestive CpGs. (XLSX 47 kb)

Additional file 5: **Table S4.** Enrichments of Minute-120 suggestive CpGs and transcription factor binding sites in gene regions. (XLSX 190 kb)

Additional file 6: **Table S5.** Summary of samples and WGBS sequencing. (XLSX 43 kb)

## Abbreviations

5mC: 5-methylcytosine
AHB: Africanized honey bee
Cf2: Chorion factor 2
CGRP: calcitonin gene-related peptide
CpG: Cytosine-guanine dinucleotide
CRF: corticotropin-releasing factor
DEG: Differentially expressed gene
Dfr: Drifter gene
DH31-R: Diuretic hormone 31 receptor
DH44: Diuretic hormone 44
DMR: Differentially methylated region
DNA: Deoxyribonucleic acid
DNMT: DNA methyltransferase
EHB: European honey bee
FDR: False discovery rate
Fkh: Forkhead gene
foxo: forkhead box, sub-group O
H3K27ac: histone H3 lysine 27 acetylation
Hr51: Hormone receptor 51 gene
IL-6: Interleukin 6
Jigr1: Jing interacting regulatory gene 1
Kb: Kilobase
L-LTP: Late phase of long-term potentiation
Lola: longitudinals lacking gene
MB: Mushroom body
NMDA: N-methyl-D-aspartate
NMDR3: N-methyl-D-aspartate receptor 3
Sbf: SET domain binding factor
SET: Su(var)3–9, Enhancer-of-zeste and Trithorax
SLC6A4: solute carrier family 6 member 4
Tet: Ten-Eleven Translocation
TFBS: Transcription factor binding site
TRP channels: Transient receptor potential channels
Vvl: Ventral veins lacking gene
WGBS: Whole-genome bisulfite sequencing
Zif: Zinc finger protein
