# Supplementary figures and images for: Defense against territorial intrusion is associated with DNA methylation changes in the honey bee brain
Source: BMC Genomics. 2018 Mar 26;19:216. doi: 10.1186/s12864-018-4594-0 (PMC5870497; doi:10.1186/s12864-018-4594-0)

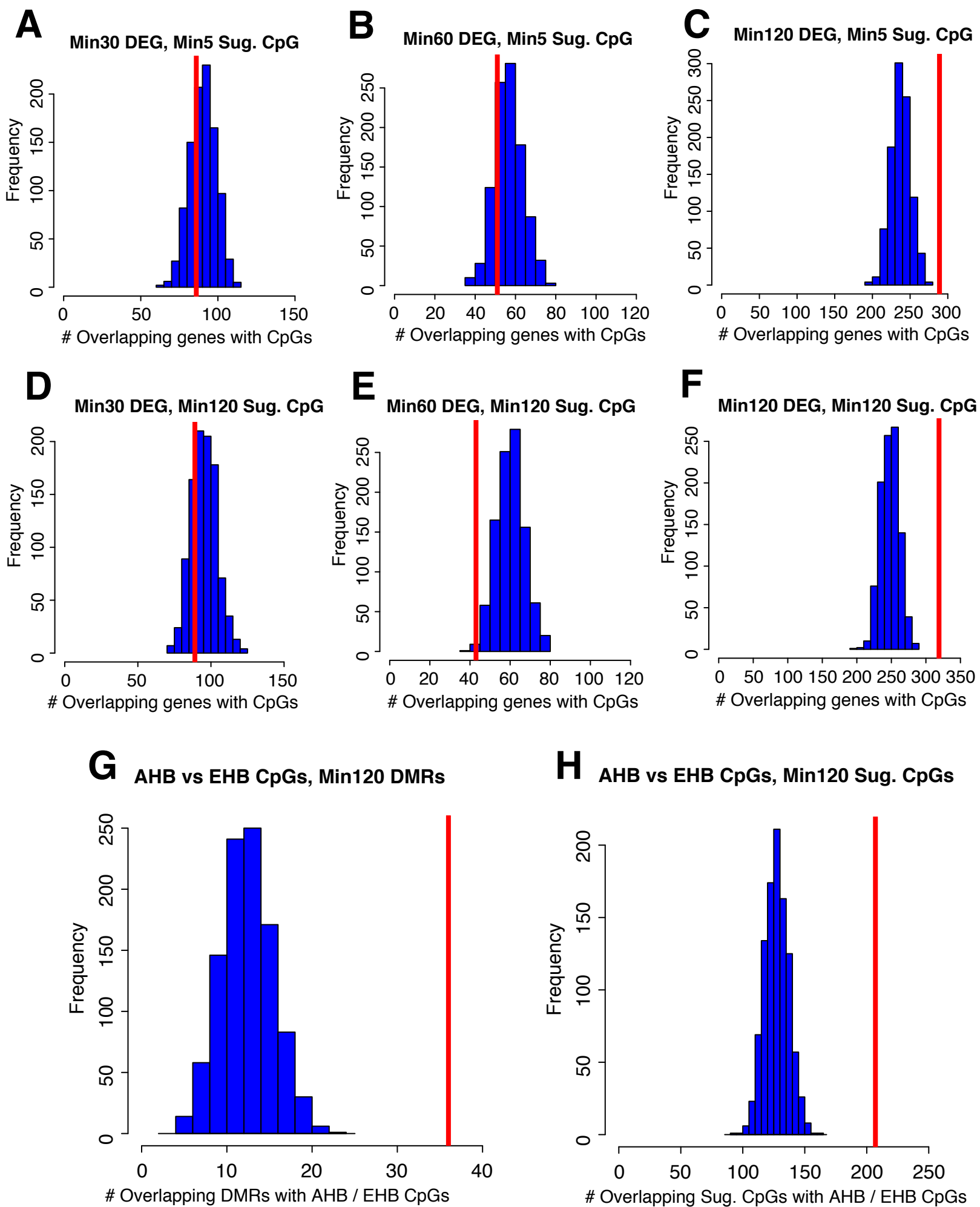

Supplement: Supplementary file 3 — Figure S1. Results of permutation testing for DEG/CpG overlap and comparison with Cingolani et al. 2013 [9]. (a-f) Minute 5 and Minute 120 suggestive CpGs were overlapped with gene expression differences of Aggressive and Control bees from Shpigler et al. (2017a) [4]. To test the significance of the overlap, we chose at random the same number of significantly different DEGs, checked the overlap and repeated this process 1000 times. For each plot, the blue histogram represents the distribution of random trials and the red vertical line represents the true number of overlapping DEGs with suggestive CpGs. Note that both minute 5 and minute 120 suggestive CpGs had a highly significant overlap with only minute 120 DEGs. (g-h) A similar approach was taken to test the significance of the overlap between AHB / EHB differences and minute 120 DMRs and suggestive CpGs. In both cases the true overlap was greater than the 1000 permutations. (PDF 478 kb) [file 12864_2018_4594_MOESM3_ESM.pdf]
